# Some Points in Diphtheria

**Published:** 1881-06

**Authors:** A. T. Conley

**Affiliations:** Cannon Falls, Minn.


### ARTICLE III.

"SOME POINTS IN DIPHTHERIA." By A. T. CONLEY, M.D., Cannon Falls, Minn.

In the April number of THE JOURNAL AND EXAMINER is a paper by Dr. C. J. Lewis on "Some Points in Diphtheria." During nine months of last year I saw and treated over one hundred cases of diphtheria, and from my experience I must take issue with the Doctor on one or two of his points. I am fully aware that different epidemics of that disease differ widely in many symptoms, and this may be one reason that his experience is so different from mine.

The first point I wish to notice is this: The Doctor says, "I am of the opinion that diphtheria is not produced by germs, nor do I believe it to be contagious." Whether diphtheria or any other contagious disease is caused by germs, is not the point I wish to discuss, for at the best, in our present state of research and knowledge, we have little else but fine-spun theories. But that there is a specific cause for each and all of the so-called contagious diseases, seems reasonable from the nature of things, and that this specific cause, whatever it is, can be and is communicated from one person to another, is as clearly proven as any other fact that rests on observation and reason; and that diphtheria conducts itself like other contagious diseases, observation and experience fully attest. One illustration from many I could give will suffice. Last fall in one of the departments of our public school a little girl came down with diphtheria and was kept in school all day. All the schools were closed from that day, but in less than a week eight other cases came down, and all children who were at school the day the little girl came down, and *all in the same department;* and though there were four other departments in the same building, not a child in any of them came down.

I have further noticed that where one came down with the disease in a family of children, every other child in the family was almost invariably sure to have it; and, further, where strict quarantine was enforced among the *well* and sick alike, that is, where children, though well, were kept strictly at home, *in no single case did such children have the disease.* Another point where my observations seem to differ from other observers is this: Though we had about two hundred cases in our town and vicinity, other physicians noting carefully with myself, in no case has the disease occurred twice in the same individual.

I have gone through epidemics of scarlet fever and other contagious diseases, and I cannot avoid the conclusion that diphtheria, for children, is the most contagious disease I have ever seen, excepting, perhaps, small-pox.

The Doctor gives a very condensed history of two cases. From the facts given I cannot see how he could positively diagnosticate diphtheria. To me it would have been probable had the disease been in the family and other cases been better marked. I am sure I never saw a case result from *cold.*

The second point: The Doctor says, "To me diphtheria is a pyrexia—an inflammatory fever, closely allied to rheumatism." Admitting for the moment that the argument adduced is correct, would not the same line of reasoning prove that scarlet fever or croup are inflammatory fevers closely allied to rheumatism? Again, the Doctor tells us that "pain is a prominent symptom in diphtheria." "That the pain caused in the fauces by attempts to swallow liquids is out of all proportion to the apparent local lesion." These statements are exactly the opposite of my experience. I do not remember to have ever heard a child crying with pain or show in any way that it was suffering severe pain. I have seen the membrane thick and heavy, and so much pushed up into the mouth that it could be seen if the mouth were opened ever so little. In fact the membrane had the appearance, as near as anything I can compare it to, of a pancake rolled into a cylinder and thrust down the throat; and yet medicine, water and milk could be swallowed with an ease that to me was amazing. Occasionally there was a case of paralysis of the muscles of deglutition; stiffness of the neck and swelling of the glands were pretty constant symptoms. Again, the Doctor says, "Epidemics of diphtheria follow periods when rheumatism has prevailed." This was certainly not the case with us; for years rheumatism has not been a prominent disease in these parts, and yet the epidemic of diphtheria has been terribly severe. Rheumatism seldom attacks children under five years old. In our epidemic more children under five years had diphtheria than over that age. The majority of cases of rheumatism occur between the ages of 15 and 30. I have seen but a very few cases of diphtheria in persons over 15 years of age. Then in rheumatism we have the rheumatic diathesis, and it is often hereditary. Where is our diphtheretic diathesis and heredity? Rheumatism, though a very painful disease, is seldom fatal, though it may last for weeks and months. Diphtheria, not a painful disease, is very fatal, often proving so in a very few days. I fail to see the close alliance between diphtheria and rheumatism.

One feature in some of my cases was that the little patients did not complain much in any way, and were often up and dressed until a short time before death. I will give one case in point. August 2, 1880, call in the country to see a little Swede girl, five years old. Found my patient running around the house; tonsils and fauces heavily covered with membrane; glands of the neck much swollen; a profuse discharge from the nose; very offensive breath; vomiting a little; no appetite; dull and congested countenance; pulse 120; bowels and kidneys reported normal by the parents. After prescribing for this patient, and going to my buggy, some fifteen rods from the house, I found, on looking round, my patient with half a dozen other children, at my heels. This child died in less than 48 hours, without obstruction to respiration.

This is not an exceptional case. Where children had the best of care and attention they often showed a disposition to be up and would play if allowed to. This occurred often in cases that run a rapid course and terminated fatally in five or six days. I frequently had much difficulty to convince my neighboring medical friends whom I called in, that these cases were in danger.

Other cases, however, were depressed from the first and the countenance had a dull leaden hue, the eyes were languid and expressionless, taking but little interest or notice of anything, but always answering when spoken to.

In discussing this subject I have aimed to give observations and symptoms and not theories. I have no pet theories to advance either as to the ætiology or treatment of diphtheria. I have no faith in the sulphites. I have used various atomizers with carbolic acid, sulphite of sodium, bi-carbonate of sodium, lime water, etc.; results not very satisfactory. Quinia and stimulants used often and early I do believe in. Monsell's solution with carbolic acid applied carefully to the membrane, I am sure is a good remedy, also cold water applied early to the neck. Chlorate of potassium I like, used in the first stages. Tincture chloride of iron in my hands has not come up to my expectations. I believe the best and surest remedy in the world is to keep the children entirely away from the disease.

April 30, 1881.

---

DR. VON NUSSBAUM, of Munich, has performed his three hundredth ovariotomy.—*British Medical Journal.*

SIR WILLIAM JENNER, K.C.B., F.R.S., was elected President of the Royal College of Physicians, April 11, in the room of Sir J. Risdon Bennett, M.D., F.R.S.—*Ibid.*

